# Retraction: The effect and molecular mechanism of hypoxia on proliferation and apoptosis of CD133+ renal stem cells

**DOI:** 10.17305/bb.2023.9285

**Published:** 2023-08-01



The Editorial Board of Biomolecules and Biomedicine has decided to retract the article titled “The effect and molecular mechanism of hypoxia on proliferation and apoptosis of CD133+ renal stem cells” (Bosn J Basic Med Sci 2021 Jun 1;21(3):313–22. doi: 10.17305/bjbms.2020.4887) following the authorsʼ request [1].

The authors have informed us that some of the original data supporting this study was inadvertently lost. Upon re-conducting the crucial experiments, they found that the trend of CD133 as depicted in Figure 1C did not align with the results initially reported in the manuscript.

In their statement, the authors noted: “After the loss of some original data, we repeated the experiments and discovered discrepancies when grouping by age; the trend of CD133 in Figure 1C did not replicate the results in the original text (see attachment). Subsequent experiments could not validate the original conclusion that the concentration of CD133+ in renal stem cells peaks in the neonatal group and decreases with age. As a result, we acknowledge there are inaccuracies in the scientific content of the study. To prevent any potential confusion or misinterpretation among readers and to maintain the credibility of the journal, we request a retraction in line with the journalʼs policy.”

Acknowledging the authorsʼ transparency, and in adherence with our journalʼs retraction policy, the decision to retract the paper was made. We regret any misunderstanding or inconvenience this may have caused to our readers. We commend the authors for their dedication to scientific precision and their responsible approach to this issue.

Biomolecules and Biomedicine remains firmly committed to upholding the highest standards of research integrity. We assure the readers that we will continue to take all necessary steps to rectify the scientific record when warranted.

## References

[1] Liu H, Liu C, Qu Y (2021 Jun). The effect and molecular mechanism of hypoxia on proliferation and apoptosis of CD133+ renal stem cells. Bosn J Basic Med Sci.

